# Effective imaging examination evaluation method for surgical pathological complete responds of head and neck squamous cell carcinoma after neoadjuvant immunochemotherapy

**DOI:** 10.3389/fonc.2025.1585194

**Published:** 2025-06-10

**Authors:** Yudong Ning, Yixuan Song, Yuqin He, Han Li, Yang Liu, Shaoyan Liu

**Affiliations:** Department of Head and Neck Surgical Oncology, National Cancer Center/National Clinical Research Center for Cancer/Cancer Hospital, Chinese Academy of Medical Sciences and Peking Union Medical College, Beijing, China

**Keywords:** pathological complete response, neoadjuvant immunotherapy, head neck squamous carcinoma, immune checkpoint inhibitors, imaging examination, response evaluation criteria in solid tumors, region of interest

## Abstract

**Objective:**

To explore an effective method for imaging examinations to evaluate the surgical pathological complete response (PCR) in patients with head and neck squamous cell carcinoma (HNSCC) following neoadjuvant immunochemotherapy (NIC).

**Methods:**

HNSCC patients who underwent NIC and subsequent surgery from May 2021 to November 2024 were retrospectively analyzed. All patients underwent imaging examination evaluations, including enhanced computed tomography (CT) and enhanced magnetic resonance (MR) imaging both before and after NIC. The average value of the region of interest (ROI) was extracted from the imaging examinations. Clinical parameter-related data were collected. The paired chi-square test was performed to analyze the differences in complete response (CR) between imaging examinations and pathology according to the response evaluation criteria in solid Tumors version 1.1 (RECISTv1.1). The optimal cutoff values of the adaptive ROI average value were determined using receiver operating characteristic curves (ROC). Binary logistic regression was applied to analyze the relevant clinical factors of PCR.

**Results:**

In total, data from 81 patients with enhanced CT and enhanced MR were included in this study. Significant discrepancies in CR were observed between enhanced CT, MRI, and pathology (21.0% *vs* 42.0%, 8.6% *vs* 42.0%) (P < 0.05). The ROI average value ratio (before/after NIC) was associated with a better PCR. Specifically, ROI average value ratio ≥ 1.18 on enhanced CT (odds ratio [OR] 125.306,95% confidence interval [CI] 5.545-2831.633,P <0.001; PCR 80.6% *vs* 11.1%) or ROI value ratio ≥ 1.06 on T2-weighted image of enhanced MR (OR 144.822,95%CI 9.271-2262.326,P < 0.001; PCR 90.3% *vs* 12.0%) was noted.

**Conclusion:**

Based on RECIST v 1.1, discrepancies in PCR were found between imaging examinations and surgical pathology of HNSCC after NIC. The ROI average value ratio (before/after NIC) was associated with a better PCR, with an enhanced CT ROI average value ratio ≥ 1.18 or the ROI average value ratio ≥ 1.06. Thus, RECIST v1.1 was demonstrated to be an inaccurate assessment method for PCR in HNSCC after NIC. The ROI average value ratio may have good diagnostic efficacy for PCR in HNSCC patients receiving NIC.

## 1 Introduction

Head and neck Squamous cell carcinoma(HNSCC) is a common malignant tumor (1). Surgery and radiotherapy constitute the primary treatment modalities for HNSCC patients. Regrettably, the vast majority of patients present at the middle or late stages of the disease at the time of treatment. Even after undergoing a series of comprehensive therapies including surgery, radiotherapy, and chemotherapy, the recurrence and mortality rates remain distressingly high (2–4). Tumor immunotherapy has emerged as a novel and promising approach in cancer treatment (5, 6). Immune checkpoint inhibitors (ICIs), such as those targeting Programmed death ligand-1 (PD-1), Programmed death ligand (PD-L1), and Cytotoxic T lymphocyte-associated protein 4 (CTLA–4), have been approved for the treatment of various cancer types, including melanoma, lung cancer, and HNSCC (7–11). Evidenced by the clinical trials of Keynotes-012, KEYNOTE-040, KEYNOTE-048, and CheckMate141, ICIs have demonstrated the ability to extend the overall survival of patients with recurrent/metastatic HNSCC (12–15). Nivolumab and pembrolizumab were approved by the U.S. Food and Drug Administration (FDA) in 2016 for use as first-line treatments for recurrent/metastatic HNSCC. Inspired by these encouraging outcomes, numerous centers have initiated explorations into neoadjuvant immunotherapy for locally advanced HNSCC, yielding satisfactory results. After neoadjuvant immunotherapy, a significant number of patients achieve a favorable pathological response, and in some cases, even a pathological complete response (PCR) (16–18). Accurate assessment of PCR is of utmost importance as it can potentially spare patients from major surgical trauma. As a non-invasive examination method, imaging is widely utilized to evaluate the efficacy of immunotherapy. However, precisely evaluating this remains a challenge for radiologists (19, 20). Currently, the Response evaluation criteria in solid tumors version 1.1 (RECIST v1.1) serves as the primary reference for the objective evaluation standard of immunotherapy efficacy in clinical trials (21).

Notwithstanding, the accuracy of RECIST v1.1, as reported in the literature, has been a subject of contention. In the literature surveyed, all relevant trials adopted RECIST v1.1 as the evaluation standard. Nine trials demonstrated a correlation between pathological and radiological responses (18, 22–29), while six trials reported discrepancies (17, 30–34). Building on our own cases, this study employed surgical pathology as the gold standard to verify the accuracy of conventional imaging methods, such as enhanced computed tomography (CT) and enhanced magnetic resonance (MR), in evaluating the PCR of neoadjuvant immunochemotherapy (NIC) for HNSCC based on RECIST v1.1. Additionally, the study aimed to identify effective imaging parameters for the accurate evaluation of PCR, with the ultimate goal of better guiding clinical practice.

## 2 Methods

### 2.1 Characteristics of the cohort

A total of 81 patients who underwent enhanced CT and MR were retrospectively analyzed. These patients were all diagnosed with HNSCC in stage III or IV, received NIC, and then underwent surgery at the head and neck surgery department of the National Cancer Center/National Clinical Research Center for Cancer/Cancer Hospital, Chinese Academy of Medical Sciences and Peking Union Medical College, from May 2021 to November 2024. Patient data, including gender, age, tumor type, differentiation, presence of multiple cancers, TNM staging, types of ICIs, and the number of immunotherapy cycles, were collected. TNM staging was determined according to the eighth edition of the staging system of The American Joint Committee on Cancer (AJCC). The clinical information of all patients was comprehensively documented.

### 2.2 Inclusion and exclusion criteria

**Inclusion Criteria:**

- All patients had locally advanced HNSCC in stage III or IV HNSCC, regardless of resectability, and underwent surgery following NIC.
- All patients underwent enhanced CT or MR imaging.
- All patients had complete medical records.

**Exclusion Criteria:**

- Patients with distant metastatic HNSCC.
- Patients who were unable to tolerate surgery.
- Patients with incomplete imaging evaluations.
- Patients with incomplete medical records.

### 2.3 Diagnostic evaluation

#### 2.3.1 Pathology evaluation

Before NIC, a biopsy of the primary tumor sites was obtained from all patients, and their cancer was pathologically confirmed as squamous cell carcinoma. Pathologically, PCR was defined as the absence of tumor cells in the resected tissue.

#### 2.3.2 Enhanced CT and MR examination

All patients underwent a 64-slice spiral CT scan (LightSpeed; VCT or Discovery HD750; GE Healthcare, USA). After a plain CT scan, patients were administered contrast media (1.2 mL/kg; Omnipaque 350 mg I/mL; GE Healthcare, USA) at a flow rate of 3.5 mL/s, followed by the injection of 40 mL of saline solution into the elbow vein using a power syringe (Medrad Stellant, Indianola, PA) at a flow rate of 3.0 mL/s. The arterial phase was captured 15 s after the injection of the contrast agent, and the venous phase was captured 45 s later. Images were then acquired.

The MRI scan was carried out using a 3.0T scanner (GE Discovery MR 750, GE Medical Systems) equipped with an 8 - channel head and neck phased - array coil. Axial Fast Spoiled Gradient Recalled (FSPGR) contrast - enhanced T1-weighted imaging (T1WI) was performed 60 s after an intravenous injection of gadopentetate dimeglumine (Magnevist, Bayer, Leverkusen, Germany) at a dose of 0.2 ml/kg body weight and a flow rate of 1.5 ml/s.

**Figure 1:**
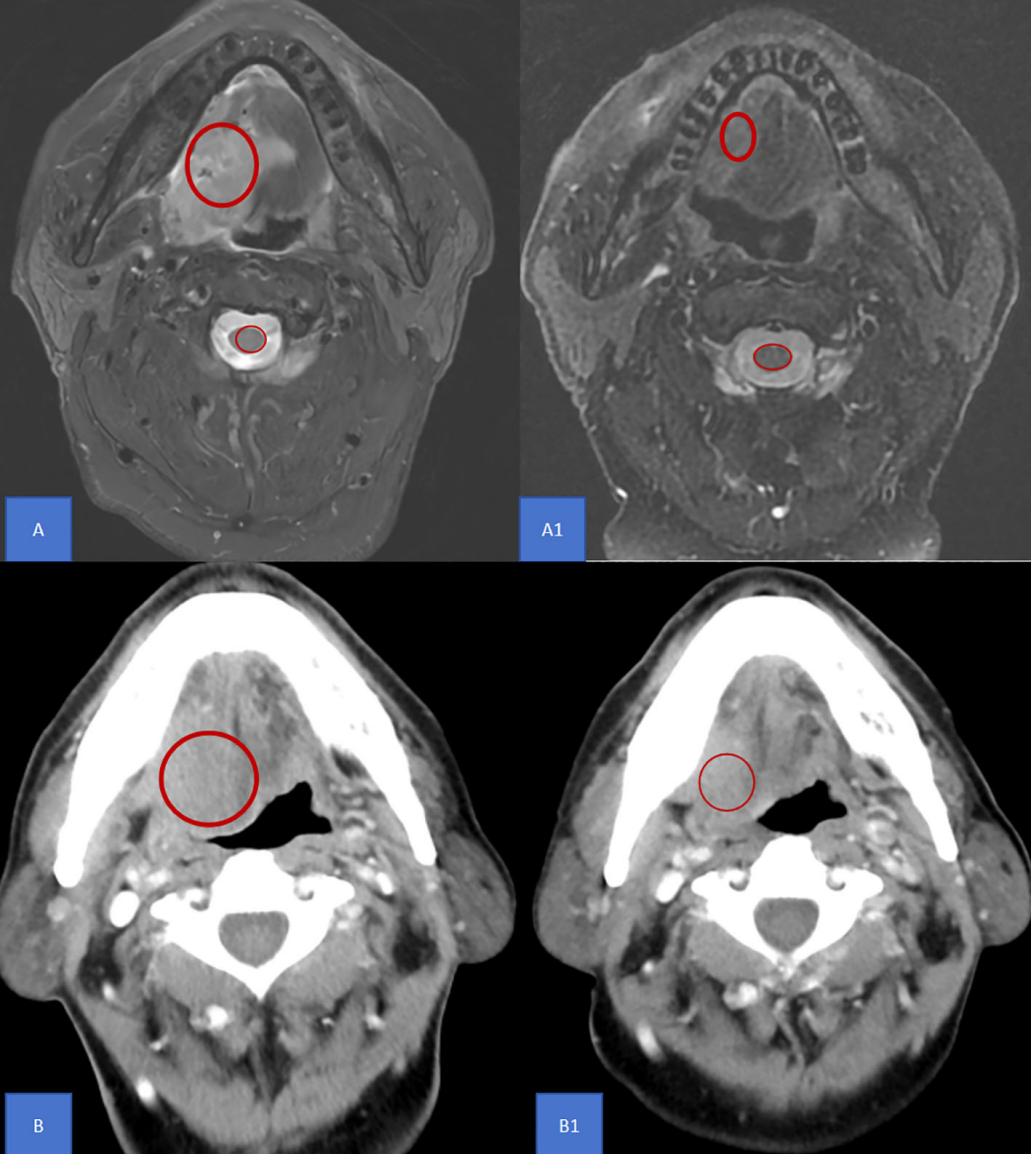
ROI definition The ROI average value was extracted from the maximum cross-sectional area of the tumor using a picture archiving and communication system as shown in the red circle. **(A)** A1 For enhanced MR, the ROI average value ratio of the T2-weighted image was calculated as the ratio of the relative ROI average value before NIC to that after NIC. The relative ROI average value was defined as the ratio of the ROI average value of the tumor to that of the spinal cord. **(B)** B1 For enhanced CT, the ROI average value ratio was calculated as the ratio of the ROI average value before NIC to that after NIC. MR, Magnetic resonance; CT, Computed tomography; AUC, Area under curve; ROI, Region of Interest; NIC, Neoadjuvant Immunochemotherapy.

All patients underwent imaging examinations, including enhanced CT or enhanced MR, both before and after NIC, to determine the clinical stage and obtain the average value of the region of interest (ROI). In imaging, the ROI refers to a specific area defined in medical images that contains important diagnostic and treatment-relevant image features. The ROI average value was extracted from the maximum cross-sectional area of the tumor using a picture archiving and communication system(PACS)(**Figure 1**). For enhanced CT, the ROI average value ratio was calculated as the ratio of the ROI average value before NIC to that after NIC. For enhanced MR, the ROI average value ratio of the T2-weighted image was calculated as the ratio of the relative ROI average value before NIC to that after NIC. The relative ROI average value was defined as the ratio of the ROI average value of the tumor to that of the spinal cord. All imaging reports were reviewed by two experienced radiologists. Radiologically, based on RECIST v 1.1, the tumor remission rate was evaluated through imaging examination. Complete response (CR) was defined as the absence of any visible tumor during imaging or pathological examination.

### 2.4 Treatment method

All patients received standard clinical treatment. They all underwent NICC, followed by surgery. NICC consisted of 200 mg of programmed death protein - 1 (PD-1) inhibitors (such as pembrolizumab, tislelizumab, toripalimab, or sintilimab) combined with cisplatin at a dose of 75 mg/m² and paclitaxel at a dose of 175 mg/m². This regimen was administered on the first day of each 21-day cycle. Surgery involved the resection of the primary tumor sites and neck dissection, with or without repair and reconstruction.

### 2.5 Statistical method

The pairwise paired chi-square test was used to analyze the differences in enhanced CT-based CR and enhanced MR-based CR between imaging examination and pathology. When 20% or more of the cells in a contingency table had an expected count of 5 or less, Fisher’s exact test was applied instead. The relationship between PCR and enhanced CT remission, enhanced MR remission based on RECIST 1.1, and the ROI average value ratio was analyzed using the receiver operating characteristic (ROC) curve, to determine the optimal cutoff values. Binary logistic regression was performed to analyze the relevant clinical factors of PCR, with odds ratios (OR) and 95% confidence intervals (CI) calculated. Statistical significance was set at P < 0.05. All statistical analyses were conducted using SPSS 27.0 software.

## 3 Results

### 3.1 Patient characteristics

A total of 81 patients who underwent enhanced CT and MR for HNSCC were included in this study (**Table 1**). The patients were stratified into two groups according to gender and age (60 years old as the cut-off). Based on tumor types, they were further divided into four subgroups: oropharyngeal cancer (20 cases, accounting for 24.7%), hypopharyngeal cancer (36 cases, 44.4%), oral cancer (17 cases, 21.0%), and laryngeal cancer (8 case, 9.9%). The number of patients with multiple cancers in this cohort was 7 (8.6%). According to pathological differentiation, the cohorts were classified into well-differentiated tumor (19 cases, 23.5%), moderately differentiated tumor (44 cases, 54.3%), and poorly differentiated tumor(18 cases, 22.2%). TNM staging results included staging III (10 cases, 12.3%) and staging IV (71 cases, 87.7%). Based on the cutoff value 1 and 20 of combined positive score(CPS),all patients were divided into three groups including CPS<1(8,9.6%),1≤CPS<20(33,40.7) and 20≤CPS(33,40.7).The patients were also categorized into four groups according to the ICIs types: pembrolizumab (43 cases, 53.1%), tislelizumab(16 cases, 19.8%), toripalimab (20 cases, 24.7%), sintilimab (2 cases, 2.5%). Based on the number of immunotherapy cycles, the patients were grouped as follows: 1 cycle (4 cases, 4.9%), 2 cycles (57 cases, 70.4%), 3 cycles (20 cases, 24.7%).

**Table 1:** Characteristics of the Cohort.

Clinical Characters	Number(%)
Gender	
Male	75(92.6)
Female	6(7.4)
Age<60	
No	38(46.9)
Yes	43(53.1)
Tumor Types	
Oropharyngeal cancer	20(24.7)
Hypopharyngeal cancer	36(44.4)
Oral cancer	17(21.0)
Laryngeal cancer	8(9.9)
Multiple Cancer	
No	74(91.4)
Yes	7(8.6)
Differentiation	
Well-differentiated	19(23.5)
Moderately differentiated	44(54.3)
Poorly differentiated	18(22.2)
TNM Staging	
III	10(12.3)
IV	71(87.7)
CPS	
CPS<1	8(9.6)
1≤CPS<20	33(40.7)
20≤CPS	40(49.4)
ICIs types	
Pabolizumab	43(53.1)
Tislelizumab	16(19.8)
Toripalimab	20(24.7)
Sintilimab	2(2.5)
Immunotherapy cycle	
1	4(4.9)
2	57(70.4)
3	20(24.7)
Total	81(100)

ICIs, immune checkpoint inhibitors; CPS, combined positive score.

### 3.2 The inaccuracy of imaging examination in evaluating surgical PCR of patients with HNSCC after NIC

**Figure 2:**
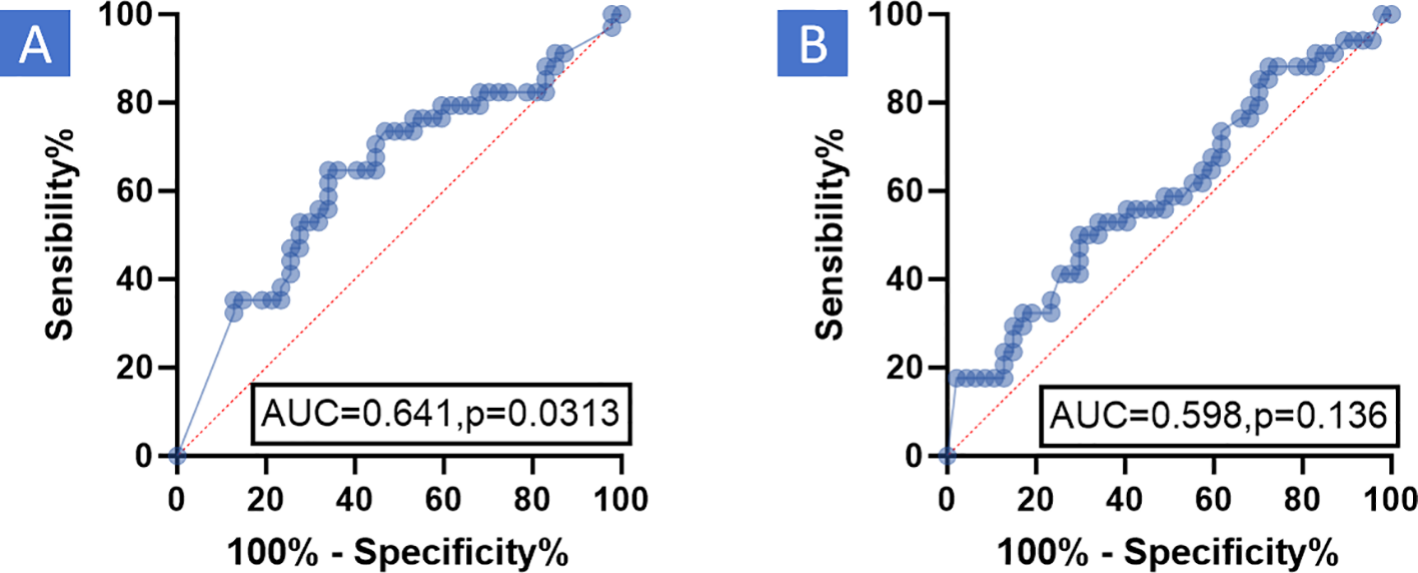
ROC the relationship between PCR and enhanced CT-based remission as well as enhanced MR-based remission, according to RECIST v 1.1, was analyzed using the Receiver Operating Characteristic (ROC) curve. The results demonstrated that enhanced CT-based remission and enhanced MR-based remission, based on RECIST v 1.1, had poor diagnostic efficacy for PCR. **(A)** Enhanced CT: AUC was 0.641, P = 0.0313. **(B)** Enhanced MR: AUC was 0.598, P = 0.136. RECIST v1.1, Response evaluation criteria in solid tumors version 1.1; MR, Magnetic resonance; PCR, Pathological complete response; CT, Computed tomography; AUC, Area under curve; ROC, Receiver operating characteristic curve.

**Figure 3:**
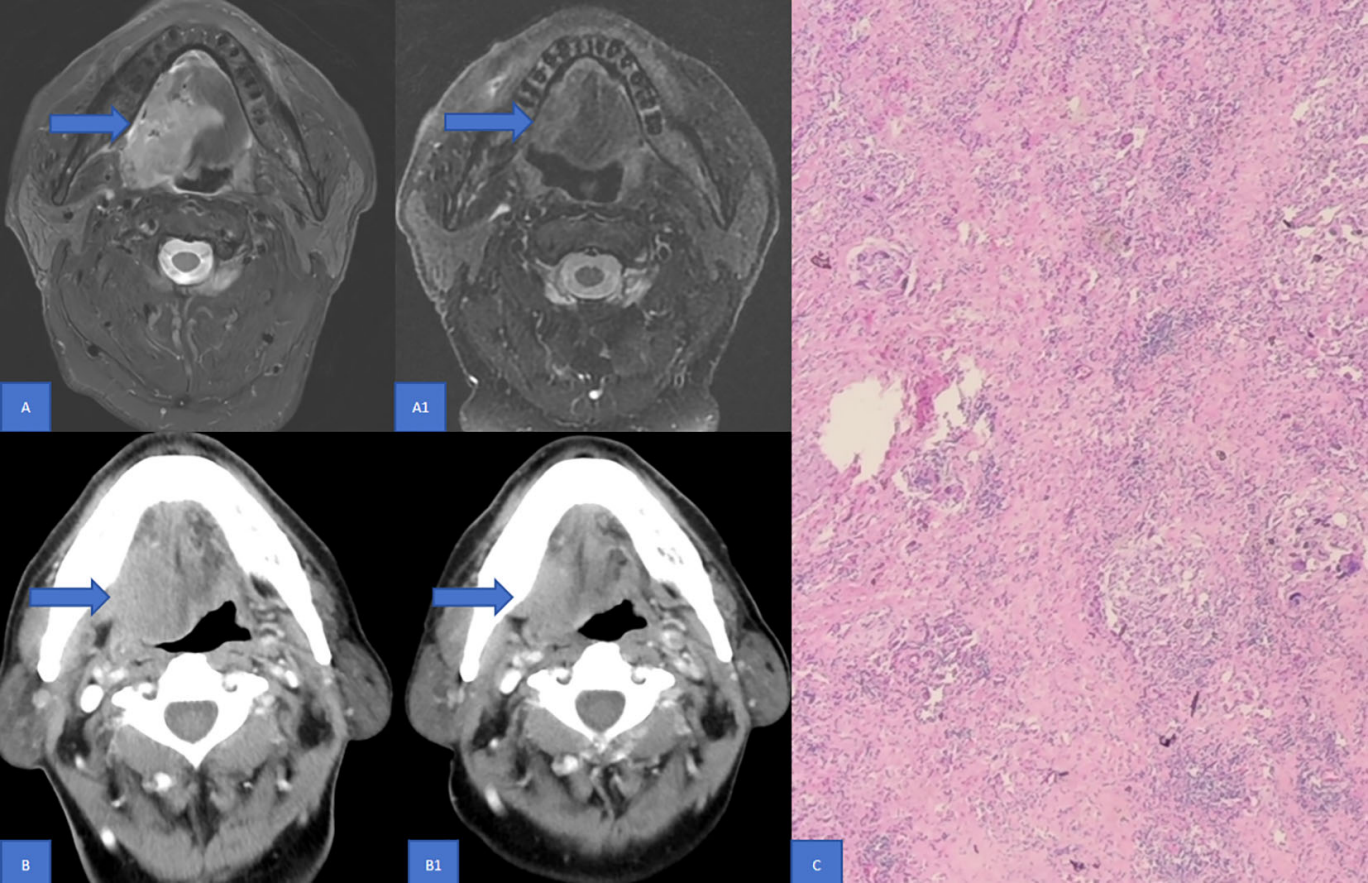
Discordance in CR between Imaging examinations and surgical pathology of HNSCC following NIC. This figure illustrates a case of tongue squamous cell carcinoma. The patient underwent two cycles of NIC and subsequent surgery. Enhanced MRI Findings: Prior to NICC, enhanced MRI disclosed an abnormal - signal mass on the left side of the tongue. It exhibited an irregular shape with a maximum cross - sectional area of approximately 5.5×3.8 cm **(A)**. Post - NICC, the enhanced MRI showed an abnormal - signal shadow on the left side of the tongue. The shape remained irregular, with a reduced size and an inhomogeneous signal. The maximum cross - section measured around 4.3×2.1 cm (A1), indicating an improvement after NIC compared to the pre-treatment state. Enhanced CT Findings: Before NIC, enhanced CT demonstrated a lesion on the left side of the tongue with an irregular configuration and a maximum cross - sectional area of roughly 5.3×3.0 cm **(B)**. After NIC, enhanced CT revealed a left - sided mass in the tongue. It was smaller than its pre - treatment size, with ill - defined boundaries, and the current dimensions were approximately 4.5×3.2 cm (B1). Pathological Outcome: The postoperative pathology report confirmed PCR **(C)**. MR, Magnetic resonance; PCR, Pathological complete response; CT, Computed tomography; CR, Complete response; NIC, Neoadjuvant immunochemotherapy.

Significant discrepancies in CR were observed between imaging examinations and pathology in both enhanced CT (21.0% *vs* 42.0%) and MR (8.6% *vs* 42.0%) (**Table 2**, P < 0.001). The relationship between PCR and enhanced CT-based remission as well as enhanced MR-based remission, according to RECIST v1.1, was analyzed using the ROC curve. It was found that enhanced CT-based remission and enhanced MR-based remission according to RECIST v1.1 had poor diagnostic efficacy for PCR, with AUC being 0.641 (**Figure 2A**, P = 0.0313) and 0.598 (**Figure 2B**, P = 0.136) respectively. **Figure 3** illustrated the discordant CR between imaging examinations and surgical pathology of HNSCC after NIC. For instance, a case of tongue squamous cell carcinoma underwent surgery after two-cycles of NIC. The postoperative pathology report indicated PCR (C), while the imaging examination still suggested the presence of a tumor (A1, B1). Before NICC, enhanced MRI showed an abnormal-signal mass on the left side of the tongue, with an irregular shape and a maximum cross - sectional area of approximately 5.5×3.8 cm (A). After NIC, enhanced MRI showed an abnormal - signal shadow on the left side of the tongue, with an irregular shape, a smaller size, and an uneven signal. The maximum cross-section was about 4.3×2.1 cm (A1), suggesting an improvement after NIC. Before NIC, enhanced CT showed a lesion on the left side of the tongue with an irregular shape and a maximum cross - sectional area of approximately 5.3×3.0 cm (B). After NIC, enhanced CT revealed a left - sided mass in the tongue, smaller than before, with unclear boundaries, and the current size was about 4.5×3.2 cm (B1).

**Table 2:** Discordant PCR between imaging examination and surgical pathology of HNSCC after NIC.

Clinical characters	Imaging examination(%)	Pathology(%)	P Value
Enhanced CT			
Non CR	64(79.0)	47(58.0)	0.001
CR	17(21.0)	34(42.0)	
Enhanced MR			
Non CR	74(91.4)	47(58.0)	<0.001
CR	7(8.6)	34(42.0)	

HNSCC, Head and Neck Squamous Cell Carcinoma; NICC, Neoadjuvant Immunotherapy Combined with Chemotherapy; PCR, pathology complete response; CT, computed tomography; MR, magnetic resonance; CR, complete response.

### 3.3 The effective index of imaging examination in evaluating surgical PCR of patients with HNSCC after NIC

**Figure 4:**
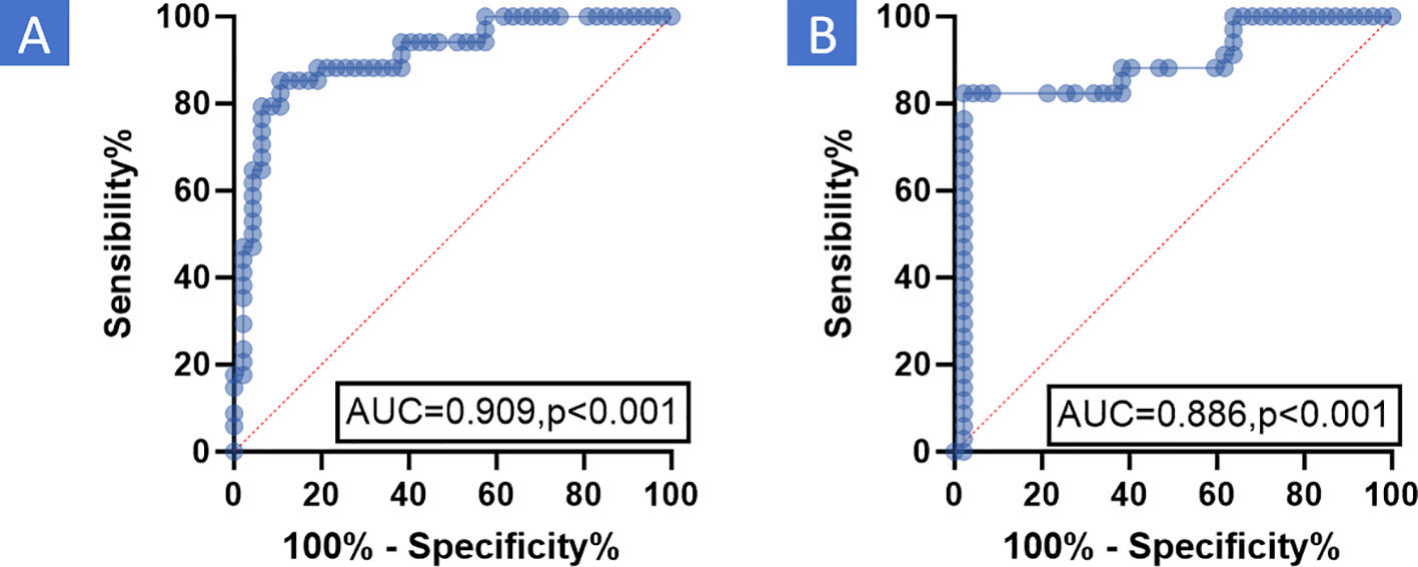
ROC the relationship between PCR and ROI average value ratio was analyzed using ROC curve to determine the effective cutoff value. The results indicated that the ROI average value ratio had good diagnostic efficacy for PCR. **(A)** Enhanced CT: For enhanced CT, the ROI average value ratio (before/after NIC) had an AUC of 0.909 and a cutoff value of 1.18 (P <0.001). **(B)** Enhanced MR: For enhanced MR, the ROI average value ratio (before/after NIC) on the T2-weighted image had an AUC of 0.886 and a cutoff value of 1.06 (P <0.001). MR, Magnetic resonance; PCR, Pathological complete response; CT, Computed tomography; AUC, Area under curve; ROC, Receiver operating characteristic Curve; ROI, Region of Interest; NIC, Neoadjuvant Immunochemotherapy.

The relationship between PCR and the ROI average value ratio was analyzed using the ROC curve to determine the optimal cutoff value. It was discovered that the ROI average value ratio had good diagnostic efficacy for PCR. For enhanced CT, the ROI average value ratio (before/after NIC) had an AUC of 0.909 and a cutoff value of 1.18 (**Figure 4A**, P <0.001). For enhanced MR, the ROI average value ratio (before/after NIC) on the T2-weighted image had an AUC of 0.889 and a cutoff value of 1.06 (**Figure 4B**, P<0.001). Binary logistic regression was performed on data including gender, age, tumor types, multiple cancers, differentiation, TNM staging, CPS, ICIs types, immunotherapy cycles, and the ROI average value ratio to analyze the relevant clinical factors of PCR (**Table 3**). Through univariate analysis, a higher PCR was significantly associated with hypopharyngeal cancer (OR 0.179 95%CI 0.055-0.589, P = 0.005),moderately differentiated tumor(OR 0.302 95%CI 0.055-0.589, P = 0.036), ROI average value ratio(before/after NIC)≥ 1.18 (OR 33.143 95% CI 9.560-114.897, P < 0.001)on enhanced CT and ROI average value ratio(before/after NIC)≥ 1.06 (OR 68.444 95% CI 15.821-296.096, P < 0.001) on enhanced MR. However, through multivariate analysis, a ROI average value ratio(before/after NIC)≥ 1.18 on enhanced CT was an indicator of a higher PCR (OR 125.306 95% CI 5.545-2831.633, P=0.002, with PCR rates of 80.6% *vs* 11.1%). In addition, through multivariate analysis, a ROI average value ratio (before/after NIC) ≥ 1.06 on the T2-weighted image was an indicator of a higher PCR (OR 144.822,95%CI 9.271-2262.326,P < 0.001; PCR 90.3% *vs* 12.0%).

**Table 3:** The univariate and multivariate analysis for PCR of clinical characteristics .

Clinical Characteristics	PCR (%)	Univariate analysis OR (95%CI)	P Value	Multivariate analysis OR(95%CI)	P Value
Gender					
Male	29(38.7)	–		–	–
Female	5(83.3)	7.931 (0.882 -71.346)	0.065	–	–
Age<60					
No	20(52.6)	–		–	–
Yes	14(32.6)	0.434 (0.176-1.070)	0.070	–	–
Tumor Types					
Oropharyngeal cancer	13(61.9)	–		–	
Hypopharyngeal cancer	9(25.0)	0.179 (0.055-0.589)	0.005	0.289 (0.019-4.468)	0.374
Oral cancer	8(50.0)	0.479 (0.127-1.798)	0.275	2.744 (0.061-124.005)	0.604
Laryngeal cancer	4(50.0)	0.538 (0.102-2.840)	0.466	4.627 (0.112-191.601)	0.420
Multiple Cancer					
No	32(43.2)	–		–	
Yes	2(28.6)	0.525 (0.096-2.883)	0.458	–	
Differentiation					
Well-differentiated	12(63.2)	–		–	–
Moderately differentiated	15(34.1)	0.302 (0.098-0.926)	0.036	0.124 (0.008-1.979)	0.124
Poorly differentiated	7(38.9)	0.371 (0.098 -1.403)	0.144	0.162 (0.006-4.414)	0.162
TNM Staging					
III	6(60.0)	–	–	–	–
IV	28(39.4)	0.434 (0.112-1.677)	0.226	–	–
CPS		–	–	–	–
CPS<1	5(31.3)	–		–	–
1≤CPS<20	14(43.8)	2.500 (0.438-14.255)	0.302		
20≤CPS	15(45.5)	2.217 (0.398-12.367)	0.346	–	–
ICIs types				–	–
Pabolizumab	20(46.5)			–	–
Tislelizumab	5(31.3)	0.523 (0.155-1.762)	0.295	–	–
Toripalimab	8(40.0)	0.767 (0.261-2.2250)	0.629	–	–
Sintilimab	1(50.0)	1.150 (0.067-19.601)	0.923		
Immunotherapy cycle		–		–	
1	2(50.0)	–		–	
2	10(38.6)	0.629 (0.082-4.792)	0.654	–	
3	10(50.0)	1.000 (0.117-8.559)	1		
ROI average value ratio(before/after NIC)on enhanced CT					
<1.18	5(11.1)	–	–	–	–
≥1.18	29(80.6)	33.143 (9.560-114.897)	<0.001	125.306 (5.545-2831.633)	0.002
ROI average value ratio(before/after NIC)on enhanced MR					
<1.06	6(12.0)				
≥1.06	28(90.3)	68.444 (15.821-296.096	<0.001	144.822(9.271-2262.326)	<0.001

CT, computed tomography; MR, magnetic resonance; PCR, pathology complete response; OR, odds ratio; CI, confidence interval; ROI, Region of interest; NIC, Neoadjuvant immunochemotherapy; ICIs, immune checkpoint inhibitors.

## 4 Discussion

ICIs represented by PD- 1/PD-L1 inhibitors are widely used in clinical immunotherapy (35, 36). In the neoadjuvant immunotherapy for HNSCC, they have achieved favorable pathological responses (37). Accurately evaluating the tumor response after immunotherapy, especially PCR, is of great significance as it can guide subsequent treatment decisions, particularly in avoiding unnecessary major surgical trauma. RECIST v1.1 is designed to quantitatively and objectively assess the treatment response following systemic therapy (21). However, in the context of immunotherapy, these criteria have been found to be insufficient (38). As a result, they have been extensively modified and repeatedly adjusted to accurately evaluate the response to different immunotherapy approaches. Immune-modified response evaluation criteria in solid tumors (iRECIST) was developed for this purpose. It shares the same criteria for lesion selection and response evaluation as RECIST v1.1, except that it requires confirmatory radiological follow-up at 4–8 weeks (39). Despite these efforts, all of these assessment methods remain complex and challenging to apply in routine clinical practice. A large-scale meta-analysis explored the impact of iRECIST on RECIST v 1.1 and found that implementing iRECIST did not significantly affect response-related endpoints (40). Currently, RECIST v1.1 is still commonly used, but its evaluation value, especially in assessing the immune response after NIC in HNSCC, is controversial, and there are relatively few relevant studies.

In our study, we retrospectively analyzed a cohort of 81 patients who underwent enhanced CT and MR. All patients had stage III or IV HNSCC, received NIC, and then underwent surgery at our center. The pairwise paired chi-square test was used to analyze the differences in CR between enhanced CT and MR imaging examinations and pathology. The relationship between PCR and enhanced CT-based remission as well as enhanced MR-based remission, according to RECIST v1.1, was analyzed using the ROC curve. Significantly discordant CR rates were observed between imaging examinations and pathology in both enhanced CT and MR (**Table 2**, P < 0.001). The ROC curve analysis revealed that enhanced CT-based remission and enhanced MR-based remission according to RECIST v1.1 had poor diagnostic efficacy for PCR (**Figures 2A, B**, P >0.05). **Figure 3** illustrated the discordant CR between imaging examinations and surgical pathology of HNSCC after NIC. These findings suggest that RECIST v1.1 is an inaccurate method for assessing the immune response in HNSCC after NIC. This is consistent with current research on neoadjuvant immunotherapy for HNSCC, which indicates that RECIST v1.1, based on enhanced CT and enhanced MR, underestimates the frequency and depth of pathological responses after neoadjuvant immunotherapy (18, 28, 41, 42). In existing guidelines, tumor response assessment mainly relies on the size of the tumor in medical images. However, it is now widely acknowledged that the lumps visible in medical images consist not only of cancer cells but also of a diverse range of non-cancer cells (43), such as immune cells, blood vessel cells, lymphocytes, fat cells, and fibroblasts. Moreover, in the context of immunotherapy, tumors may exhibit atypical response patterns that are difficult to describe using traditional criteria. These unique response patterns include pseudoprogression, hyperprogression, dissociation response, and persistent response, as demonstrated by radiographic examples (19, 39, 44). This further emphasizes the need to identify effective evaluation modalities or criteria after neoadjuvant immunotherapy.

Radiomics involves extracting a large number of features from medical imaging data using data - characterization algorithms to define tumor patterns and features that are not visible to the naked eye (45). A study has shown that, based on the CT features of non - small cell lung cancer and melanoma patients undergoing immunotherapy, lesions with uneven morphological features, compact boundaries, and heterogeneous density patterns are associated with a response to immunotherapy (46). According to MR imaging, the field of radiomics may also provide additional information for prognostic prediction of immunotherapy based on established molecular biomarkers (47).

The definition of ROI is a concept commonly used in 3D image processing to extract a specific region in an image for further analysis. A detailed analysis of the tumor is crucial for understanding tumor heterogeneity, but this information is often difficult to obtain due to the spatial resolution and contrast limitations of images. Radiomic features, such as those related to ROI, can extract this information because they represent physiologically distinct regions with different blood flow, cell density, and water conditions. Changes in these regions may help predict treatment outcomes (48). Chen et al (49). compared the radiomic features of tumors and peritumoral tissues within different ROI ranges and found that the radiomic features extracted from the tumor and the 10-millimeter peritumoral region had the best predictive ability in predicting the response of liver cancer to the first transarterial chemoembolization therapy. In our study, we utilized the simple and accessible ROI approach to improve the diagnostic efficiency of PCR for HNSCC after NIC through conventional imaging examinations such as enhanced CT and MR. The relationship between PCR and the enhanced CT and enhanced MR based on the ROI average value ratio was analyzed using the ROC curve, and effective cutoff values were determined. The ROI average value ratio (before/after NIC) was associated with a better PCR. Specifically, ROI average value ratio ≥ 1.18 on enhanced CT (odds ratio [OR] 125.306,95% confidence interval [CI] 5.545-2831.633, P <0.001; PCR 80.6% *vs* 11.1%) or ROI value ratio ≥ 1.06 on T2-weighted image of enhanced MR (OR 144.822,95%CI 9.271-2262.326,P < 0.001; PCR 90.3% *vs* 12.0%) was noted.

Although radiomics is a promising tool for evaluating and predicting immune efficacy by mining more data than traditional data, it has not yet been applied in daily clinical practice (50). In fact, most studies have been based on small groups of patients, mostly from one institution. In addition, they are observational and retrospective in design, resulting in a lack of standardization of image acquisition protocols. Another notable methodological flaw in the current study is the lack of adequate external validation, which is intrinsic to the stability of the radiological model. Therefore, radiological models whose radiological signatures show different critical values in different studies cannot be widely generalized (51). Fluorine [18F] deoxyglucose positron emission tomography/computed tomography imaging (18F - FDG - PET/CT) has shown promise in the context of immunotherapy. It can characterize response patterns, assess treatment response using metabolic response criteria, and even provide patient prognostic information, mainly in patients with non - small cell lung cancer and advanced melanoma (52). However, its high cost limits its widespread use.

The complexity of monitoring tumor response in patients treated with ICIs has spurred the development of novel radiotracers. In particular, the PET/CT PD - L1 tracers currently used in clinical research have a strong correlation with PD-L1 status as measured by immunohistochemistry (53). Moreover, these tracers can reveal the heterogeneity of PD-L1 expression between different patients and within tumor lesions in the same patient on PET/CT, even more accurately than immunohistochemically stained biopsy samples (53, 54). Our center participated in a study reporting a 68GA - labeled targeted covalent radiopharmaceutical fibroblast - activating protein inhibitor (68Ga- TCR - FAPI) that demonstrated enhanced and sustained tumor targeting. It has shown significant clinical value in medullary thyroid carcinoma, and its clinical translational value in evaluating tumor immune-efficacy is yet to be fully explored (55). However, to our knowledge, only a few imaging probes are currently in the clinical research stage and have not been approved by the FDA for clinical use (56).

The novelty and originality of our study are as follows. First, our team demonstrated the inaccuracy of RECIST v1.1 in evaluating NIC in HNSCC. Second, we used the simple and accessible ROI method to improve the diagnostic efficiency of PCR for HNSCC after NIC through conventional imaging examinations such as enhanced CT and MR.

Nevertheless, our study has certain limitations. First, the overall sample size was small, with a predominance of oropharyngeal and hypopharyngeal cancers, while the number of oral and laryngeal cancer cases was limited. This imbalance may have introduced potential bias, and thus, large - sample - size or multi - center studies are needed for validation. Second, the heterogeneity in the types of immunological agents used could lead to variability in the study results. Third, our study had a retrospective design, which may also introduce some bias. Therefore, in the future, large - scale, prospective studies are required to further confirm our findings.

## 5 Conclusion

In conclusion, our study comprehensively investigated the evaluation of surgical PCR in HNSCC patients following NIC. By comparing imaging examinations with surgical pathology based on RECIST 1.1, significant discordances in PCR were detected. Specifically, the rates of CR determined by enhanced CT and MRI imaging examinations were markedly lower than those verified by surgical pathology. This discrepancy clearly demonstrates that RECIST 1.1 is an inaccurate assessment method for PCR in HNSCC after NIC.

On the other hand, we explored the potential of ROI average value ratio (before/after NIC) as an alternative evaluation index. Our findings indicated that a ROI average value ratio ≥ 1.18 on enhanced CT or a ROI average value ratio ≥ 1.06 on the T2-weighted image of enhanced MR was associated with a better PCR. This suggests that the ROI average value ratio may possess good diagnostic efficacy for PCR in HNSCC patients undergoing NIC.

Overall, these results highlight the limitations of current response evaluation criteria in the context of immunotherapy for HNSCC and propose a potentially more effective imaging-based parameter for assessing PCR. Future research should focus on validating these findings in larger cohorts and exploring the underlying mechanisms to further improve the accuracy of evaluating the efficacy of NIC in HNSCC.

## Data Availability

The raw data supporting the conclusions of this article will be made available by the authors, without undue reservation.

## References

[1] Johnson DE Burtness B Leemans CR Lui VWY Bauman JE Grandis JR. Head and neck squamous cell carcinoma. Nat Rev Dis Primers. (2020) 6:92. doi: 10.1038/s41572-020-00224-3 33243986 PMC7944998

[2] Bernier J Domenge C Ozsahin M Matuszewska K Lefebvre JL Greiner RH. Postoperative irradiation with or without concomitant chemotherapy for locally advanced head and neck cancer. N Engl J Med. (2004) 350:1945–52. doi: 10.1056/NEJMoa032641 15128894

[3] Sexton GP Walsh P Moriarty F O'Neill JP. The changing face of Irish head and neck cancer epidemiology: 20 years of data. Eur Arch Otorhinolaryngol. (2022) 279:3079–88. doi: 10.1007/s00405-021-07118-4 PMC9072499 34647138

[4] Cramer JD Burtness B Le QT Ferris RL. The changing therapeutic landscape of head and neck cancer. Nat Rev Clin Oncol. (2019) 16:669–83. doi: 10.1038/s41571-019-0227-z 31189965

[5] Meng W Huang L Guo J Xin Q Liu J Hu Y. Innovative nanomedicine delivery: targeting tumor microenvironment to defeat drug resistance. Pharmaceutics. (2024) 16:1–24. doi: 10.3390/pharmaceutics16121549 PMC11728492 39771528

[6] Ning Y Li H Song Y He Y Liu S Liu Y. Predictive value of CPS combined with inflammatory markers for pathological remission of locally advanced head and neck squamous cell carcinoma after adjuvant immunochemotherapy. Front Mol Biosci. (2025) 12:1593742. doi: 10.3389/fmolb.2025.1593742 40376264 PMC12078134

[7] Borghaei H Paz-Ares L Horn L Spigel DR Steins M Ready NE. Nivolumab versus Docetaxel in Advanced Nonsquamous Non-Small-Cell Lung Cancer. N Engl J Med. (2015) 373:1627–39. doi: 10.1056/NEJMoa1507643 PMC5705936 26412456

[8] Brahmer J Reckamp KL Baas P Crino L Eberhardt WE Poddubskaya E. Nivolumab versus Docetaxel in Advanced Squamous-Cell Non-Small-Cell Lung Cancer. N Engl J Med. (2015) 373:123–35. doi: 10.1056/NEJMoa1504627 PMC4681400 26028407

[9] Herbst RS Baas P Kim DW Felip E Perez-Gracia JL Han JY. Pembrolizumab versus docetaxel for previously treated, PD-L1-positive, advanced non-small-cell lung cancer (KEYNOTE-010): a randomised controlled trial. Lancet. (2016) 387:1540–50. doi: 10.1016/S0140-6736(15)01281-7 26712084

[10] Long GV Weber JS Larkin J Atkinson V Grob JJ SChadendorf D. nivolumab for patients with advanced melanoma treated beyond progression: analysis of 2 phase 3 clinical trials. JAMA Oncol. (2017) 3:1511–9. doi: 10.1001/jamaoncol.2017.1588 PMC5710191 28662232

[11] Postow MA Chesney J Pavlick AC Robert C Grossmann K McDermott D. Nivolumab and ipilimumab versus ipilimumab in untreated melanoma. N Engl J Med. (2015) 372:2006–17. doi: 10.1056/NEJMoa1414428 PMC5744258 25891304

[12] Seiwert TY Burtness B Mehra R Weiss J Berger R Eder JP. Safety and clinical activity of pembrolizumab for treatment of recurrent or metastatic squamous cell carcinoma of the head and neck (KEYNOTE-012): an open-label, multicentre, phase 1b trial. Lancet Oncol. (2016) 17:956–65. doi: 10.1016/S1470-2045(16)30066-3 27247226

[13] Cohen EEW Soulieres D Le Tourneau C Dinis J Licitra L Ahn MJ. Pembrolizumab versus methotrexate, docetaxel, or cetuximab for recurrent or metastatic head-and-neck squamous cell carcinoma (KEYNOTE-040): a randomised, open-label, phase 3 study. Lancet. (2019) 393:156–67. doi: 10.1016/S0140-6736(18)31999-8 30509740

[14] Harrington KJ Ferris RL Blumenschein G Jr. Colevas AD Fayette J Licitra L. Nivolumab versus standard, single-agent therapy of investigator's choice in recurrent or metastatic squamous cell carcinoma of the head and neck (CheckMate 141): health-related quality-of-life results from a randomised, phase 3 trial. Lancet Oncol. (2017) 18:1104–15. doi: 10.1016/S1470-2045(17)30421-7 PMC6461049 28651929

[15] Burtness B Harrington KJ Greil R Soulieres D Tahara M de Castro G Jr.. Pembrolizumab alone or with chemotherapy versus cetuximab with chemotherapy for recurrent or metastatic squamous cell carcinoma of the head and neck (KEYNOTE-048): a randomised, open-label, phase 3 study. Lancet. (2019) 394:1915–28. doi: 10.1016/S0140-6736(19)32591-7 31679945

[16] Leidner R Crittenden M Young K Xiao H Wu Y Couey MA. Neoadjuvant immunoradiotherapy results in high rate of complete pathological response and clinical to pathological downstaging in locally advanced head and neck squamous cell carcinoma. J Immunother Cancer. (2021) 9:1–25. doi: 10.1136/jitc-2021-002485 PMC8108690 33963014

[17] Ferris RL Spanos WC Leidner R Goncalves A Martens UM Kyi C. Neoadjuvant nivolumab for patients with resectable HPV-positive and HPV-negative squamous cell carcinomas of the head and neck in the CheckMate 358 trial. J Immunother Cancer. (2021) 9:1–11. doi: 10.1136/jitc-2021-002568 PMC8183204 34083421

[18] Vos JL Elbers JBW Krijgsman O Traets JJH Qiao X van der Leun AM. Neoadjuvant immunotherapy with nivolumab and ipilimumab induces major pathological responses in patients with head and neck squamous cell carcinoma. Nat Commun. (2021) 12:7348. doi: 10.1038/s41467-021-26472-9 34937871 PMC8695578

[19] Borcoman E Kanjanapan Y Champiat S Kato S Servois V Kurzrock R. Novel patterns of response under immunotherapy. Ann Oncol. (2019) 30:385–96. doi: 10.1093/annonc/mdz003 30657859

[20] Nishino M Hatabu H Hodi FS. Imaging of cancer immunotherapy: current approaches and future directions. Radiology. (2019) 290:9–22. doi: 10.1148/radiol.2018181349 30457485 PMC6312436

[21] Eisenhauer EA Therasse P Bogaerts J Schwartz LH Sargent D Ford R. New response evaluation criteria in solid tumours: revised RECIST guideline (version 1.1). Eur J Cancer. (2009) 45:228–47. doi: 10.1016/j.ejca.2008.10.026 19097774

[22] Huang X Liu Q Zhong G Peng Y Liu Y Liang L. Neoadjuvant toripalimab combined with gemcitabine and cisplatin in resectable locally advanced head and neck squamous cell carcinoma (NeoTGP01): An open label, single-arm, phase Ib clinical trial. J Exp Clin Cancer Res. (2022) 41:300. doi: 10.1186/s13046-022-02510-2 36224603 PMC9558942

[23] Knochelmann HM Horton JD Liu S Armeson K Kaczmar JM Wyatt MM. Neoadjuvant presurgical PD-1 inhibition in oral cavity squamous cell carcinoma. Cell Rep Med. (2021) 2:100426. doi: 10.1016/j.xcrm.2021.100426 34755137 PMC8561313

[24] Ferrarotto R Bell D Rubin ML Hutcheson KA Johnson JM Goepfert RP. Impact of Neoadjuvant Durvalumab with or without Tremelimumab on CD8(+) Tumor Lymphocyte Density, Safety, and Efficacy in Patients with Oropharynx Cancer: CIAO Trial Results. Clin Cancer Res. (2020) 26:3211–9. doi: 10.1158/1078-0432.CCR-19-3977 PMC8362306 32269052

[25] Huang Y Sun J Li J Zhu D Dong M Dou S. Neoadjuvant immunochemotherapy for locally advanced resectable oral squamous cell carcinoma: a prospective single-arm trial (Illuminate Trial). Int J Surg. (2023) 109:2220–7. doi: 10.1097/JS9.0000000000000489 PMC10442116 37288582

[26] Luginbuhl AJ Johnson JM Harshyne LA Linnenbach AJ Shukla SK Alnemri A. tadalafil enhances immune signatures in response to neoadjuvant nivolumab in resectable head and neck squamous cell carcinoma. Clin Cancer Res. (2022) 28:915–27. doi: 10.1158/1078-0432.CCR-21-1816 PMC8898272 34911681

[27] Ju WT Xia RH Zhu DW Dou SJ Zhu GP Dong MJ. A pilot study of neoadjuvant combination of anti-PD-1 camrelizumab and VEGFR2 inhibitor apatinib for locally advanced resectable oral squamous cell carcinoma. Nat Commun. (2022) 13:5378. doi: 10.1038/s41467-022-33080-8 36104359 PMC9472189

[28] Schoenfeld JD Hanna GJ Jo VY Rawal B Chen YH Catalano PS. neoadjuvant nivolumab or nivolumab plus ipilimumab in untreated oral cavity squamous cell carcinoma: A phase 2 open-label randomized clinical trial. JAMA Oncol. (2020) 6:1563–70. doi: 10.1001/jamaoncol.2020.2955 PMC7453348 32852531

[29] Darragh LB Knitz MM Hu J Clambey ET Backus J Dumit A. A phase I/Ib trial and biological correlate analysis of neoadjuvant SBRT with single-dose durvalumab in HPV-unrelated locally advanced HNSCC. Nat Cancer. (2022) 3:1300–17. doi: 10.1038/s43018-022-00450-6 PMC9701140 36434392

[30] Zhang Z Wu B Peng G Xiao G Huang J Ding Q. neoadjuvant chemoimmunotherapy for the treatment of locally advanced head and neck squamous cell carcinoma: A single-arm phase 2 clinical trial. Clin Cancer Res. (2022) 28:3268–76. doi: 10.1158/1078-0432.CCR-22-0666 PMC9662919 35766967

[31] Hanna GJ O'Neill A Shin KY Wong K Jo VY Quinn CT. neoadjuvant and adjuvant nivolumab and lirilumab in patients with recurrent, resectable squamous cell carcinoma of the head and neck. Clin Cancer Res. (2022) 28:468–78. doi: 10.1158/1078-0432.CCR-21-2635 PMC9401515 34667025

[32] Patel SA Gibson MK Deal A Sheth S Heiling H Johnson SM. A phase 2 study of neoadjuvant chemotherapy plus durvalumab in resectable locally advanced head and neck squamous cell carcinoma. Cancer. (2023) 129:3381–9. doi: 10.1002/cncr.v129.21 37395170

[33] Redman JM Friedman J Robbins Y Sievers C Yang X Lassoued W. Enhanced neoepitope-specific immunity following neoadjuvant PD-L1 and TGF-beta blockade in HPV-unrelated head and neck cancer. J Clin Invest. (2022) 132:1–13. doi: 10.1172/JCI172059 PMC9479764 35727629

[34] Uppaluri R Campbell KM Egloff AM Zolkind P Skidmore ZL Nussenbaum B. neoadjuvant and adjuvant pembrolizumab in resectable locally advanced, human papillomavirus-unrelated head and neck cancer: A multicenter, phase II trial. Clin Cancer Res. (2020) 26:5140–52. doi: 10.1158/1078-0432.CCR-20-1695 PMC7547532 32665297

[35] Wang DR Wu XL Sun YL. Therapeutic targets and biomarkers of tumor immunotherapy: response versus non-response. Signal Transduct Target Ther. (2022) 7:331. doi: 10.1038/s41392-022-01136-2 36123348 PMC9485144

[36] Meng WJ Guo JM Huang L Zhang YY Zhu YT Tang LS. Anoikis-related long non-coding RNA signatures to predict prognosis and immune infiltration of gastric cancer. Bioengineering (Basel). (2024) 11:1–14. doi: 10.3390/bioengineering11090893 PMC11428253 39329635

[37] Nindra U Hurwitz J Forstner D Chin V Gallagher R Liu J. A systematic review of neoadjuvant and definitive immunotherapy in locally advanced head and neck squamous cell carcinoma. Cancer Med. (2023) 12:11234–47. doi: 10.1002/cam4.v12.10 PMC10242857 36934434

[38] Ning YD Song YX He YQ Li H Liu SY. Discordant responses between imaging examination and surgical pathology of head and heck squamous cell carcinoma after neoadjuvant immunotherapy combined with chemotherapy. World J Oncol. (2025) 16:59–69. doi: 10.14740/wjon1973 39850520 PMC11750755

[39] Seymour L Bogaerts J Perrone A Ford R Schwartz LH Mandrekar S. iRECIST: guidelines for response criteria for use in trials testing immunotherapeutics. Lancet Oncol. (2017) 18:e143–52. doi: 10.1016/S1470-2045(17)30074-8 PMC5648544 28271869

[40] Kataoka Y Hirano K. Which criteria should we use to evaluate the efficacy of immune-checkpoint inhibitors? Ann Transl Med. (2018) 6:222. doi: 10.21037/atm.2018.04.17 30023385 PMC6035987

[41] Cohen EEW Bell RB Bifulco CB Burtness B Gillison ML Harrington KJ. The Society for Immunotherapy of Cancer consensus statement on immunotherapy for the treatment of squamous cell carcinoma of the head and neck (HNSCC). J Immunother Cancer. (2019) 7:184. doi: 10.1186/s40425-019-0662-5 31307547 PMC6632213

[42] Haddad R Concha-Benavente F Blumenschein G Jr. Fayette J Guigay J Colevas AD. Nivolumab treatment beyond RECIST-defined progression in recurrent or metastatic squamous cell carcinoma of the head and neck in CheckMate 141: A subgroup analysis of a randomized phase 3 clinical trial. Cancer. (2019) 125:3208–18. doi: 10.1002/cncr.v125.18 PMC6771504 31246283

[43] Baghban R Roshangar L Jahanban-Esfahlan R Seidi K Ebrahimi-Kalan A Jaymand M. Tumor microenvironment complexity and therapeutic implications at a glance. Cell Commun Signal. (2020) 18:59. doi: 10.1186/s12964-020-0530-4 32264958 PMC7140346

[44] Kanjanapan Y Day D Wang L Al-Sawaihey H Abbas E Namini A. Hyperprogressive disease in early-phase immunotherapy trials: Clinical predictors and association with immune-related toxicities. Cancer. (2019) 125:1341–9. doi: 10.1002/cncr.31999 30768786

[45] Guo J Meng W Li Q Zheng Y Yin H Liu Y. Pretreatment sarcopenia and MRI-based radiomics to predict the response of neoadjuvant chemotherapy in triple-negative breast cancer. Bioengineering (Basel). (2024) 11:1–14.. doi: 10.3390/bioengineering11070663 PMC11274092 39061745

[46] Khorrami M Prasanna P Gupta A Patil P Velu PD Thawani R. changes in CT radiomic features associated with lymphocyte distribution predict overall survival and response to immunotherapy in non-small cell lung cancer. Cancer Immunol Res. (2020) 8:108–19. doi: 10.1158/2326-6066.CIR-19-0476 PMC7718609 31719058

[47] Kang CY Duarte SE Kim HS Kim E Park J Lee AD. artificial intelligence-based radiomics in the era of immuno-oncology. Oncologist. (2022) 27:e471–83. doi: 10.1093/oncolo/oyac036 PMC9177100 35348765

[48] Gillies RJ Kinahan PE Hricak H. Radiomics: images are more than pictures, they are data. Radiology. (2016) 278:563–77. doi: 10.1148/radiol.2015151169 PMC4734157 26579733

[49] Chen M Cao J Hu J Topatana W Li S Juengpanich S. clinical-radiomic analysis for pretreatment prediction of objective response to first transarterial chemoembolization in hepatocellular carcinoma. Liver Cancer. (2021) 10:38–51. doi: 10.1159/000512028 33708638 PMC7923935

[50] Rizzo S Botta F Raimondi S Origgi D Fanciullo C Morganti AG. Radiomics: the facts and the challenges of image analysis. Eur Radiol Exp. (2018) 2:36. doi: 10.1186/s41747-018-0068-z 30426318 PMC6234198

[51] Reuze S Schernberg A Orlhac F Sun R Chargari C Dercle L. Radiomics in nuclear medicine applied to radiation therapy: methods, pitfalls, and challenges. Int J Radiat Oncol Biol Phys. (2018) 102:1117–42. doi: 10.1016/j.ijrobp.2018.05.022 30064704

[52] Lang D Wahl G Poier N Graf S Kiesl D Lamprecht B. Impact of PET/CT for assessing response to immunotherapy-A clinical perspective. J Clin Med. (2020) 9:1–21. doi: 10.3390/jcm9113483 PMC7694130 33126715

[53] Niemeijer AL Hoekstra OS Smit EF de Langen AJ. Imaging responses to immunotherapy with novel PET tracers. J Nucl Med. (2020) 61:641–2. doi: 10.2967/jnumed.119.236158 32086241

[54] Niemeijer AN Leung D Huisman MC Bahce I Hoekstra OS van Dongen G. Whole body PD-1 and PD-L1 positron emission tomography in patients with non-small-cell lung cancer. Nat Commun. (2018) 9:4664. doi: 10.1038/s41467-018-07131-y 30405135 PMC6220188

[55] Cui XY Li Z Kong Z Liu Y Meng H Wen Z. Covalent targeted radioligands potentiate radionuclide therapy. Nature. (2024) 630:206–13. doi: 10.1038/s41586-024-07461-6 38778111

[56] McCarthy CE White JM Viola NT Gibson HM. *In vivo* imaging technologies to monitor the immune system. Front Immunol. (2020) 11:1067. doi: 10.3389/fimmu.2020.01067 32582173 PMC7280489

